# The *Vibrio parahaemolyticus *Type III Secretion Systems manipulate host cell MAPK for critical steps in pathogenesis

**DOI:** 10.1186/1471-2180-10-329

**Published:** 2010-12-30

**Authors:** Ksenia Matlawska-Wasowska, Rebecca Finn, Ana Mustel, Conor P O'Byrne, Alan W Baird, Eleanor T Coffey, Aoife Boyd

**Affiliations:** 1 Discipline of Microbiology, School of Natural Sciences, National University of Ireland, Galway, University Road, Galway, Ireland; 2 School of Agriculture, Food Science & Veterinary Medicine, Veterinary Science Centre, University College Dublin, Belfield, Dublin 4, Ireland; 3 Turku Centre for Biotechnology, Turku University and Åbo Akademi University, BioCity, Tykistokatu 6, Turku FIN-20521, Finland

## Abstract

**Background:**

*Vibrio parahaemolyticus *is a food-borne pathogen causing inflammation of the gastrointestinal epithelium. Pathogenic strains of this bacterium possess two Type III Secretion Systems (TTSS) that deliver effector proteins into host cells. In order to better understand human host cell responses to *V. parahaemolyticus*, the modulation of Mitogen Activated Protein Kinase (MAPK) activation in epithelial cells by an O3:K6 clinical isolate, RIMD2210633, was investigated. The importance of MAPK activation for the ability of the bacterium to be cytotoxic and to induce secretion of Interleukin-8 (IL-8) was determined.

**Results:**

*V. parahaemolyticus *deployed its TTSS1 to induce activation of the JNK, p38 and ERK MAPK in human epithelial cells. VP1680 was identified as the TTSS1 effector protein responsible for MAPK activation in Caco-2 cells and the activation of JNK and ERK by this protein was important in induction of host cell death. *V. parahaemolyticus *actively induced IL-8 secretion in a response mediated by TTSS1. A role for VP1680 and for the ERK signalling pathway in the stimulation of IL-8 production in epithelial cells by *V. parahaemolyticus *was established. Interestingly, TTSS2 inhibited IL-8 mRNA transcription at early stages of interaction between the bacterium and the cell.

**Conclusions:**

This study demonstrated that *V. parahaemolyticus *activates the three major MAPK signalling pathways in intestinal epithelial cells in a TTSS1-dependent manner that involves the TTSS1 effector VP1680. Furthermore VP1680 and JNK and ERK activation were needed for maximal cytotoxicity of the bacterium. It was shown that *V. parahaemolyticus *is a strong inducer of IL-8 secretion and that induction reflects a balance between the effects of TTSS1 and TTSS2. Increases in IL-8 secretion were mediated by TTSS1 and VP1680, and augmented by ERK activation. These results shed light on the mechanisms of bacterial pathogenesis mediated by TTSS and suggest significant roles for MAPK signalling during infection with *V. parahaemolyticus*.

## Background

*Vibrio parahaemolyticus *is a gram negative, halophilic bacterium that is found in warm marine environments, such as the commensal microflora of shellfish [1,2]. The bacterium is a major food-borne pathogen that causes acute gastroenteritis following consumption of undercooked or raw shellfish, especially oysters. It has become an increasingly important pathogen during the last decade as pandemic strains have emerged, most likely due to rising global temperatures and increased seafood consumption [3]. Approximately 50% of all cases of food-borne gastroenteritis in Southeast Asia are due to *V. parahaemolyticus*. It is one of the major health and economic problems in this region and the incidence of infection is rising throughout the United States, South America and Europe [4-8]. The bacterium infects the human intestinal epithelium causing diarrhoea, intestinal inflammation, abdominal cramps, nausea, vomiting, headaches, fever, chills and in some cases even death [8,9]. Intestinal epithelial responses to *V. parahaemolyticus *infection include the activation of the inflammatory cascade, infiltration of phagocytes, epithelial cell damage, alterations in the structure and function of the tight junction barrier and the induction of fluid and electrolyte secretion [10,11].

Sequencing of the genome of a pandemic strain of *V. parahaemolyticus *(RIMD2210633) in 2003 revealed the presence of two sets of genes encoding two separate Type III Secretion Systems, named TTSS1 and TTSS2 [12]. TTSS1 is present in all *V. parahaemolyticus *strains and is involved in host cell cytotoxicity, while TTSS2 is responsible for enterotoxicity (the ability to induce fluid accumulation in the intestine) and is predominantly found in pathogenic strains [13-15]. More recently a third TTSS, that is closely related to TTSS2, was identified in *trh*-positive pathogenic strains of *V. parahaemolyticus *[16]. TTSS effector proteins are injected from the cytosol of bacterium directly into the cytoplasm of the host cell by means of a syringe-like delivery apparatus [17]. Once inside the host cells the effector proteins modify the activity of eukaryotic cell signalling pathways leading to changes in host cell behaviour that favour the colonization and persistence of bacteria in the host [18].

The Mitogen Activated Protein Kinases (MAPK) are a group of protein serine/threonine kinases that are activated in mammalian cells in response to a variety of extracellular stimuli and mediate signal transduction from the cell surface to the nucleus where they can alter the phosphorylation status of specific transcription factors [19-21]. Three major types of MAPK pathways have been reported so far in mammalian cells [19-21]. The ERK1/2 pathway is involved in cell proliferation and differentiation, whereas the JNK and p38 pathways are activated in response to stress stimuli [19-21]. The balance between factors activated by ERK, JNK and p38 determines whether the cell lives or dies [19-21]. Modification of MAPK signalling pathways by bacteria may contribute to induction of host cell death, which is an important feature of bacterial pathogenesis promoting bacterial tissue colonisation [17,22-24]. *V. parahaemolyticus *induces cell death via TTSS1 in epithelial cells and macrophages [14,25-28]. Most recently autophagic cell death has been implicated as the mechanism by which *V. parahaemolyticus *exerts its cytotoxicity [26,29]. The role of MAPK in the induction of autophagy and cell death by *V. parahaemolyticus *has not hitherto been investigated.

The *V. parahaemolyticus *VopP TTSS2 effector (also known as VopA) has been shown to inhibit MAPK signalling pathways in macrophages. It binds directly to MAPK kinases (MKK), the upstream kinases that phosphorylate the MAPK, and both prevents their activation and inhibits their activity. This it accomplishes by acetylating the catalytic loop of MKK, thereby inhibiting ATP binding [18,30]. Enteric pathogenic bacteria can elicit or suppress expression of cytokines and chemokines from host cells, often via modification of MAPK signalling pathways. Interleukin 8 (IL-8) is a chemokine secreted basolaterally by epithelial cells thus creating an IL-8 gradient responsible for migration of neutrophils to the site of infection and is a key player in the initiation of an inflammatory response. The MAPK are involved in the signal transduction pathways leading to IL-8 chemokine production [31-33]. To date there are no published data on the effect of *V. parahaemolyticus *infection on IL-8 expression.

Employing an *in vitro *model of intestinal epithelial infection we have found that *V. parahaemolyticus *induces JNK, ERK and p38 activation in human epithelial cells and that the TTSS1 effector VP1680 mediates the activation of p38 and JNK. Moreover, the MAPK activation within the host cells is associated with the cytotoxic effects exerted by *V. parahaemolyticus *and with the induction of IL-8 secretion by the bacterium. The diverse roles of MAPK signalling during infection with *V. parahaemolyticus *indicate that the bacterium may use more than one mechanism to sabotage normal cellular processes and disrupt host response to infection.

## Results

### *V. parahaemolyticus *activates the MAPK signalling pathways in intestinal epithelial cells

**Figure 1:**
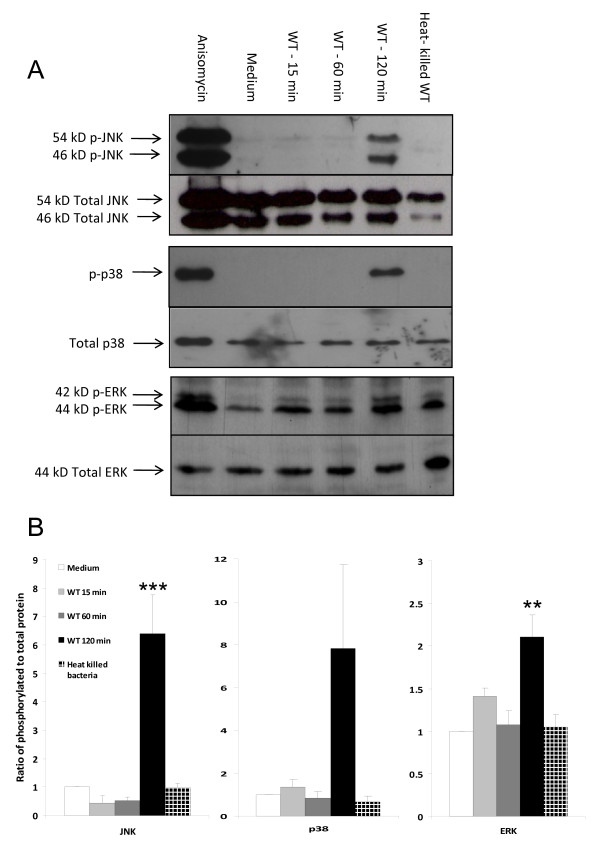
***V. parahaemolyticus *induces JNK, p38 and ERK phosphorylation in intestinal epithelial cells**. Caco-2 cells were co-incubated with viable *V. parahaemolyticus *WT RIMD2210633 for 15, 60 or 120 min, with 50 μg/ml anisomycin for 30 min or with heat-killed WT *V. parahaemolyticus *for 2 h. Cell lysates were prepared and proteins separated by SDS-PAGE. Following transfer of proteins to nitrocellulose membranes, the membranes were probed with anti-phospho-JNK, -phospho-p38 and -phospho-ERK1/2 antibodies. The stripped membranes were re-probed with the corresponding anti-total-MAPK antibodies to control for equivalent protein loading. **A**. Representative image of MAPK immunoblot. Results are representative of at least three independent experiments. **B**. Quantification of MAPK activation. Results are expressed as the ratio of phospho-MAPK to total MAPK and as relative to levels in Caco-2 cells alone. Results indicate mean ± standard error of the mean (SEM) of three independent experiments. **P < 0.01; ***P < 0.001 vs medium.

For several pathogenic bacteria modulation of the activity of the MAPK signalling pathway is a critical event in their ability to colonise the host [22-24]. The role of MAPK signalling during *V. parahaemolyticus *infection and the ability of the bacteria to modulate host cell responses via this pathway has not been elucidated so far. The first aim of our study was to examine responses of cell signalling MAPK to *V. parahaemolyticus*. Caco-2 cells were co-incubated with WT RIMD2210633 bacteria for 15, 60 and 120 min at an MOI of 10. Anisomycin was used as a positive control to induce phosphorylation of each of the MAPK. Heat-killed (60°C, 20 min) WT bacteria were included to investigate the effect of bacterial cell surface moieties on MAPK activation, in the absence of active protein synthesis and growth. The extracted proteins were subjected to immunoblotting analysis with anti-phospho-JNK, -phospho-p38 and -phospho-ERK1/2 antibodies. The stripped membranes were re-probed with anti-total-JNK, -p38, -ERK1/2 antibody to detect the total level of each MAPK protein present in the samples and to control for loading quantities. JNK and p38 were phosphorylated in cells co-incubated with the WT bacteria, in comparison to samples obtained from untreated Caco-2 cells which showed no MAPK activation (Figure 1). Strong activation of JNK and p38 was observed at the 2 h time point, but not at earlier time points. In contrast, little or no phosphorylation of JNK and p38 was detected in cells incubated for 2 h with the heat-killed WT bacteria, indicating that the induction of activation of these two MAPK is an active process of *V. parahaemolyticus *requiring viable bacteria. The patterns of ERK activation in response to *V. parahaemolyticus *were similar with lower phosphorylation signals detected. These studies indicate that *V. parahaemolyticus *induces activation of the JNK, p38 and ERK MAPK signalling pathways via a mechanism requiring metabolically active bacteria.

### TTSS1 of *V. parahaemolyticus *is responsible for activation of JNK, p38 and ERK in epithelial cells

**Figure 2:**
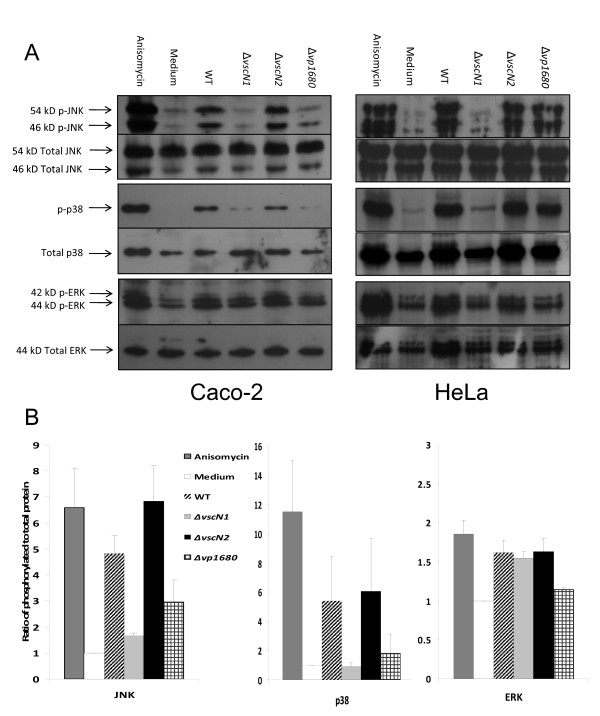
**Activation of JNK, p38 and ERK is mediated by TTSS1**. Caco-2 and HeLa cells were co-incubated with *V. parahaemolyticus *WT RIMD2210633, Δ*vscN1*, Δ*vscN2 *and Δ*vp1680 *for 2 h or with anisomycin for 30 min. Immunoblotting of cell lysates was performed as described in Figure 1. **A**. Representative image of MAPK immunoblot. Results are representative of at least three independent experiments. **B**. Quantification of MAPK activation in Caco-2 cells. Results are expressed as the ratio of phospho-MAPK to total MAPK and as relative to levels in Caco-2 cells alone. Results indicate mean ± SEM of three independent experiments.

TTSS effectors of several pathogenic bacteria have been shown to modify MAPK activation levels in eukaryotic cells [24,34-36]. As *V. parahaemolyticus *was able to induce phosphorylation of p38, JNK and ERK MAPK by an active process, we next investigated the involvement of the TTSS of *V. parahaemolyticus *in the activation of these MAPK. Bacteria lacking a functional TTSS1 or a functional TTSS2 were constructed by deleting the corresponding *vscN *gene for each secretion system. Based on homology to other TTSS the *vscN *genes are presumed to encode the ATPases that power the secretion process. Caco-2 cells were co-incubated with WT, Δ*vscN1 *and Δ*vscN2 V. parahaemolyticus *for 2 h and MAPK activation analysed by immunoblotting. Δ*vscN2 *bacteria induced similar levels of JNK phosphorylation in Caco-2 cells as those induced by the WT bacteria, when compared to untreated Caco-2 cells (Figure 2). In contrast the Δ*vscN1 *bacteria did not cause an increase in JNK activation, indicating that TTSS1 is required for the induction of JNK phosphorylation in epithelial cells by *V. parahaemolyticus*. Similarly, p38 was phosphorylated to equivalent levels in cells co-incubated with WT and Δ*vscN2 *bacteria compared to cells alone. Activation of p38 was greatly diminished when the Caco-2 cells were incubated with Δ*vscN1 *bacteria showing that the TTSS1 of *V. parahaemolyticus *plays an essential role in the activation of p38 in epithelial cells in response to infection. Conversely TTSS2 is not required for p38 or JNK activation by *V. parahaemolyticus*. The degree of ERK phosphorylation was similar in cells co-incubated with wild-type, Δ*vscN1 *and Δ*vscN2 *bacteria (Figure 2), although in each case the increase compared to cells alone was less than two-fold. As the increase in activation of ERK in Caco-2 cells was low, the ability of *V. parahaemolyticus *to induce MAPK activation in an alternative human epithelial cell line - HeLa - was investigated. There was a greater increase in the activation of ERK in response to WT bacteria in this cell line as compared to Caco-2 cells (Figure 2). The requirement for TTSS1 to activate each MAPK was evidenced by the lack of activation seen in response to the Δ*vscN1 *strain. These results provide the first evidence that activation of the JNK, p38 and ERK MAPK pathways in human epithelial cells infected with *V. parahaemolyticus *depends on the bacterium's TTSS1.

### The TTSS1-dependent cytotoxicity of *V. parahaemolyticus *succeeds MAPK activation

**Figure 3:**
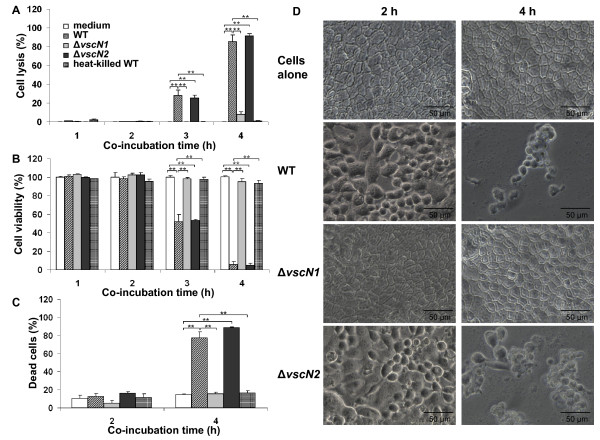
**TTSS-1 dependent cytotoxicity occurs later than MAPK activation**. Caco-2 cells were co-incubated with viable *V. parahaemolyticus *WT RIMD2210633, Δ*vscN1*, Δ*vscN2 *or with heat-killed WT *V. parahaemolyticus *for 1, 2, 3 and 4 h (A and B) or 2 and 4 h (C and D). Values are presented as mean ± SEM; **P < 0.01 vs medium and vs WT. **A: **Cell lysis was determined by assaying LDH activity in the growth medium. Results are one representative experiment performed in triplicate of three independent experiments. **B: **MTT reduction by living cells was quantified. Results, expressed as percentage of cell viability, are one representative experiment performed in triplicate of three independent experiments. **C: **Cells were stained with propidium iodide to visualize dead cells with loss of membrane integrity and with Hoechst 33342 to show nuclei in all cells. Three hundred Caco-2 cells were scored via fluorescent microscopy. The results, expressed as percentage dead cells, are from three independent experiments. **D: **Morphological changes of the Caco-2 cells were observed by phase contrast light microscope (magnification 400×).

It is well known that MAPK are activated during cellular stress responses and that they mediate signal transduction events leading to cell death. It has previously been demonstrated that *V. parahaemolyticus *induces cell death in a TTSS1-dependent manner in a variety of cell types, including Caco-2 cells. To determine whether MAPK activation in the Caco-2 cells is a consequence of the cytotoxicity of *V. parahaemolyticus *we investigated the kinetics of cytotoxicity of the bacterium in these epithelial cells. The Caco-2 monolayers were co-incubated with WT, Δ*vscN1 *and Δ*vscN2 *bacteria for 1, 2, 3 or 4 h and cytotoxicity was quantified by measurement of cell lysis (LDH assays) and cellular metabolism/viability (MTT assays). After 1 and 2 h of incubation there was no significant LDH release (Figure 3A) or decrease in cell viability (Figure 3B) observed in any of the samples. Following 3 h of incubation, WT and Δ*vscN2 V. parahaemolyticus *induced cell lysis and decreased cell viability of the Caco-2 cells in comparison to untreated cells. A dramatic increase in cell lysis and decrease in cell viability was observed in the Caco-2 cells co-incubated with the WT and Δ*vscN2 *bacteria at the 4 h time point, with more than 80% cell death. In contrast, no significant cell death was detected in samples co-incubated with the Δ*vscN1 V. parahaemolyticus *or with heat-killed WT bacteria at any time point and the levels obtained were comparable to the results obtained for untreated Caco-2 cells. Overall the results confirmed that TTSS1 is required for the cytotoxicity of *V. parahaemolyticus *towards Caco-2 cells. The LDH and MTT assay results mirrored one another, notwithstanding that MTT measures changes in cell metabolism and as such is a more sensitive reflection of cell pathology than membrane damage. Moreover, we have shown that *V. parahaemolyticus *was cytotoxic to the epithelial cells in a time-dependent manner with no cell lysis occurring at the 2 h time point and increasing amounts of cell lysis at the later 3 h and 4 h time points.

These results prompted us to determine Caco-2 cell viability using fluorochrome staining (Figure 3C). Caco-2 cells co-incubated with WT, Δ*vscN1 *and Δ*vscN2 *bacteria were stained with Hoechst 33324 to visualize cell nuclei. Propidium iodide was included in the study to visualise dead cells that incorporate the stain due to loss of their membrane integrity. The results revealed that WT *V. parahaemolyticus *and the TTSS deletion mutants did not affect the viability of the Caco-2 cells during the first 2 h of co-incubation. The cytotoxic effect of *V. parahaemolyticus *infection was observed after 4 h of incubation of the Caco-2 cells with WT and Δ*vscN2*, but not Δ*vscN1*, bacteria confirming that *V. parahaemolyticus *cytotoxicity is TTSS1-dependent.

Next we examined the morphological changes induced in epithelial cells by *V. parahaemolyticus*. Figure 3D shows the development of rounded cells after 2 h of co-incubation of the Caco-2 cells with the WT bacteria. After 4 h the rounded cells were still present but visible cell loss was also observed because of the cytotoxic effect exerted by *V. parahaemolyticus*, consistent with the LDH and MTT results. Similar to WT bacteria, the Δ*vscN2 *mutant induced cell rounding after 2 h of co-incubation and cell rounding combined with significant cell loss after 4 h. The monolayer of Caco-2 cells co-incubated with Δ*vscN1 *bacteria remained intact and exhibited the morphological features of untreated cells, even after 4 h of co-incubation, suggesting that TTSS1 is required for monolayer disruption and cell rounding and confirming its role in the cytotoxicity of *V. parahaemolyticus *towards epithelial cells.

Together these results suggest that the cytotoxicity of *V. parahaemolyticus *is TTSS1-dependent and show that this cytotoxic effect occurs after 3 h of co-incubation. As strong MAPK activation is observed after 2 h of co-incubation, we propose that MAPK activation is not a consequence of cytotoxicity, but rather it might be a prerequisite for cytotoxicity.

### JNK and ERK are involved in the TTSS1-dependent cytotoxicity of *V. parahaemolyticus*

**Figure 4:**
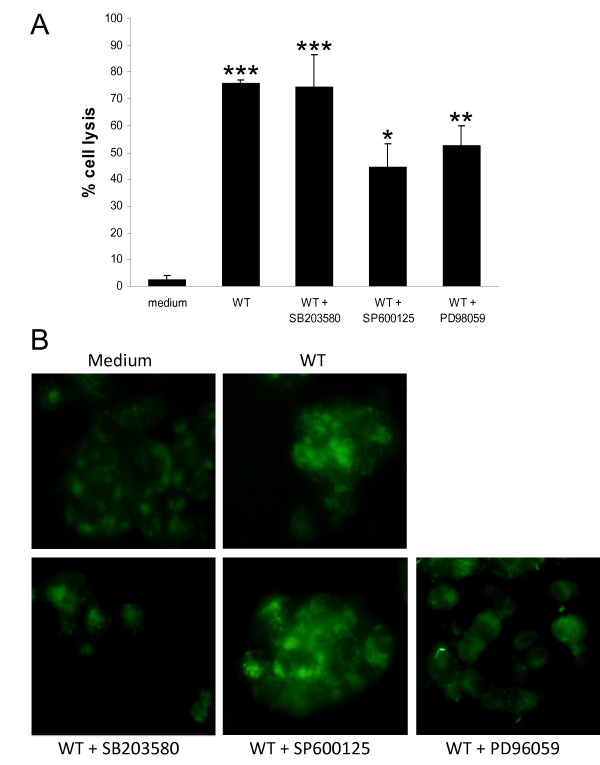
**Role of MAPK in cytotoxicity of *V. Parahaemolyticus***. Caco-2 cells were co-incubated with *V. parahaemolyticus *WT RIMD2210633 for 3 h (MDC staining) or 4 h (LDH assay), either alone or in combination with one of the MAPK inhibitors, SB203580 (5 μM), SP600125 (15 μM) or PD98059 (40 μM). **A**. LDH assays were performed to quantify cell lysis. Results indicate mean ± SEM of three independent experiments. *P < 0.05; **P < 0.01; ***P < 0.001 vs medium. **B**. MDC staining was visualised by fluorescent microscopy.

As MAPK signalling pathways are involved in cell fate determination by co-ordinately regulating a wide range of cellular activities ranging from gene expression, metabolism and motility to mitosis, survival, differentiation and apoptosis [20], we next sought to determine whether the cytotoxicity of *V. parahaemolyticus *was a result of MAPK activation by the use of MAPK inhibitors. SP600125 is a reversible ATP-competitive inhibitor of JNK that prevents the phosphorylation of JNK substrates. In an analogous manner SB203580 is a specific inhibitor of p38 by acting as a competitive inhibitor of ATP binding. PD98059 is a selective inhibitor of MEK1 activation and the ERK cascade, as it binds to the inactive forms of MEK1 and prevents activation by upstream activators. The concentration of inhibitors that abrogated MAPK activity was initially determined by titration experiments with 7-day Caco-2 cells stimulated with anisomycin. The activation levels of ERK, the p38 target MK-2 and the JNK target c-jun in cell lysates were assessed by immunoblotting with phospho-specific antibodies. Each MAPK inhibitor specifically reduced the phosphorylation of its cognate indicator protein (data not shown). To assess the importance of MAPK activation in the cytotoxic ability of *V. parahaemolyticus*, WT bacteria were co-incubated with Caco-2 cells in the presence of SB203580, SP600125 or PD98059 for 4 h and then the LDH assay was performed to quantify the level of cell lysis. The inhibitors alone did not affect the viability of the Caco-2 cells (data not shown). The JNK and ERK inhibitors (SP600125 and PD98059, respectively) caused a decrease in *Vibrio*-induced cell lysis of the Caco-2 cells. Cytotoxicity was reduced by about a third by each of these inhibitors (Figure 4A). In contrast, there was no significant difference in the level of cell lysis that occurred in samples incubated with or without the p38 inhibitor (SB203580). Addition of both SP600125 and PD98059 together during the co-incubation did not decrease cytotoxicity levels below the level seen with either inhibitor alone (data not shown). The results suggest that activation of JNK and ERK, but not p38, is involved in the ability of *V. parahaemolyticus *to be cytotoxic to the Caco-2 cells. Recently autophagic cell death has been implicated as the mode of TTSS1-mediated cytotoxicity [25,29]. The effect of the MAPK inhibitors on the induction of this process by WT *V. parahaemolyticus *was assessed by visualising monodansylcadaverine (MDC) accumulation in autophagic vacuoles. Increased MDC accumulation occurred upon co-incubation with WT bacteria (Figure 4B) and this accumulation was less evident in the presence of the ERK inhibitor PD98059. These results indicate that activation of ERK by *V. parahaemolyticus *may influence cytotoxicity at the stage of autophagy induction, while JNK may act at a later stage.

### The TTSS1 effector VP1680 regulates MAPK activation

The results above demonstrated that TTSS1 was responsible for stimulating the activation of p38 and JNK in epithelial cells in response to *V. parahaemolyticus*. Three proteins have so far been identified as TTSS1 effector proteins, namely VP1680 (also known as VopQ and VepA), VP1686 (also known as VopS) and VPA0450 and of these three proteins VP1680 has been implicated in the ability of *V. parahaemolyticus *to be cytotoxic to epithelial cells [25,29]. As we had shown a link between the two TTSS1-dependent activities of cytotoxicity and MAPK activation, the role of VP1680 in these processes was next investigated. First a strain of *V. parahaemolyticus *containing a knock-out of the *vp1680 *gene was constructed. To authenticate the mutation, the level of cell lysis induced by the Δ*vp1680 *strain was determined by the LDH assay. Over a 4 h period the viability of the Caco-2 cells co-incubated with the Δ*vp1680 *strain was comparable to the viability of cells co-incubated with the Δ*vscN1 *strain (at the 4 h time point the percentage cell lysis values were: WT - 52±8%; Δ*vscN1 *- 10±2%; Δ*vp1680 *-8±1%) confirming that the VP1680 TTSS1 effector protein is the principle factor responsible for the cytotoxicity of *V. parahaemolyticus *towards epithelial cells. Analysis of the morphology of the cells co-incubated with the Δ*vp1680 *bacteria showed that the cells were still attached to the substratum as a confluent monolayer, but appeared rounded and did not display evidence of tight junctions (Additional file 1, Figure S1). In contrast cells co-incubated with Δ*vscN1 *bacteria were indistinguishable from non-infected cells. This indicated that VP1686 [37,38] was being the translocated into host cells by Δ*vp1680 *bacteria and that TTSS1 was functional in this strain.

Analysis of the ability of this Δ*vp1680 *strain to induce MAPK activation in the Caco-2 and HeLa cells was performed by immunoblotting of the extracted proteins with anti-phospho-JNK, -p38 and -ERK antibodies (Figure 2). In Caco-2 cells, the Δ*vp1680 *strain lacked the ability to activate p38 and JNK to the extent seen with the WT, indicating that VP1680 was the TTSS1 effector required for activation of these two MAPK. While reduced ERK activation was observed with the Δ*vp1680 *strain as compared to the WT, a conclusive resolution could not be drawn, due to the low overall fold increase in ERK activation. In HeLa cells a more pronounced decreased phosphorylation of ERK occurred in response to Δ*vp1680*. In contrast, VP1680 was only partly responsible for activation of p38 and JNK, as just a slight reduction in phosphorylation was seen in cells co-incubated with the *V. parahaemolyticus *strain lacking this effector as compared to WT bacteria. As activation of the MAPK is not abolished when VP1680 is non-functional, this suggests that there is an alternative TTSS1 effector that can activate MAPK in HeLa cells, but not in Caco-2 cells

Our results show that VP1680 is necessary for the activation of JNK and p38 in Caco-2 cells and that JNK is involved in the VP1680-dependent cytotoxicity of *V. parahaemolyticus*. These data together demonstrate that VP1680 is required for the ability of *V. parahaemolyticus *to be cytotoxic to epithelial cells, at least in part through activation of JNK.

### Both TTSS are involved in modulation of IL-8 secretion by intestinal epithelial cells in response to *V. parahaemolyticus*

**Figure 5:**
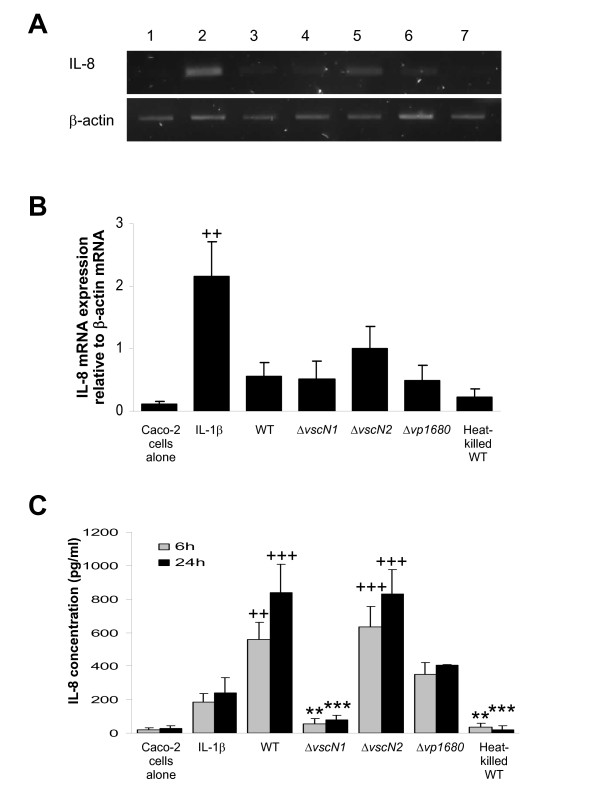
**TTSS modulate IL-8 secretion by intestinal epithelial cells in response to *V. parahaemolyticus***. **A**: IL-8 RT-PCR on cellular extracts after co-incubation with *V. parahaemolyticus*. Caco-2 cells were co-incubated for 2 h with - Lane 1: medium alone, Lane 2: 20 ηg/ml IL-1β, Lane 3: *V. parahaemolyticus *WT, Lane 4: Δ*vscN1*, Lane 5: Δ*vscN2*, lane 6: Δ*vp1680 *and lane 7: heat killed WT *V. parahaemolyticus*. RNA was extracted and reverse-transcribed. PCR amplification of IL-8, and β-actin as a control, was performed on the cDNA and visualized after migration on agarose gel by SYBRsafe staining. Results are a representative experiment of three independent experiments. **B**: Quantification of band intensity was performed on the samples described in Panel A and results are presented as the ratio between IL-8 mRNA quantification and β-actin mRNA quantification. Results indicate mean ± SEM of three independent experiments. ^++^P < 0.01 vs medium. **C**: ELISA to detect secreted IL-8 6 h and 24 h after co-incubation with *V. parahaemolyticus*. Caco-2 cells were co-incubated with *V. parahaemolyticus *WT RIMD2210633, Δ*vscN1*, Δ*vscN2*, Δ*vp1680 *and heat killed WT *V. parahaemolyticus *for 2 h. Then cells were washed with PBS and the remaining extracellular bacteria killed by addition of gentamicin. Supernatant was recovered 4 h and 22 h after that and thus 6 h and 24 h, respectively, after the beginning of the co-incubation for quantification of IL-8 by ELISA. Results indicate mean ± SEM of three independent experiments. ^++^P < 0.01; ^+++^P < 0.001 vs medium and **P < 0.01; ***P < 0.001 vs WT.

In response to pathogenic bacteria, intestinal epithelial cells produce a number of pro-inflammatory cytokines and chemokines, such as IL-8 which attracts neutrophils to the site of infection and can lead to inflammatory responses that may facilitate bacterial infection and colonisation [39-41]. The MAPK are key players in the signal transduction pathways that lead to IL-8 secretion. Therefore we tested the ability of *V. parahaemolyticus *to induce IL-8 secretion from Caco-2 cells and investigated the role of the TTSS and the MAPK in this event. The *V. parahaemolyticus *strains carrying mutations in each of the two TTSS were co-incubated with Caco-2 cells and the IL-8 response was measured by RT-PCR and ELISA (Figure 5A and 5C respectively). IL-1β was added as a positive control for the induction of IL-8 secretion. RNA extracts were prepared after 2 h of co-incubation while the supernatant used for ELISA detection of IL-8 was recovered 24 h later.

The RT-PCR results showed that IL-8 transcription was strongly activated by the IL-1β positive control and was induced to a lower extent by WT *V. parahaemolyticus*, while there was no increase of transcription observed using the heat-killed *V. parahaemolyticus *(Figure 5A). This result shows that live *V. parahaemolyticus *actively induces IL-8 transcription. The *ΔvscN1 *and Δ*vp1680 *strains induced similar levels of IL-8 transcription in the Caco-2 cells to the WT *V. parahaemolyticus*, while the *ΔvscN2 *strain induced a high level of IL-8 transcription (more than 4-fold the level of IL-8 transcript induced by the WT *V. parahaemolyticus*). This suggests that after 2 h of co-incubation TTSS1 is not involved in IL-8 mRNA production by the Caco-2 cells, while TTSS2 is involved in the inhibition of the IL-8 transcription.

The ELISA results show that 24 h after co-incubation, WT *V. parahaemolyticus *is a powerful activator of IL-8 secretion by Caco-2 cells, as there was a 15-fold increase in IL-8 concentrations after WT *V. parahaemolyticus *co-incubation in comparison to untreated Caco-2 cells (Figure 5C). Similar IL-8 concentrations were detected with the Caco-2 cells alone and in the presence of heat-killed WT *V. parahaemolyticus*. A dramatic reduction of IL-8 secretion was observed in response to *ΔvscN1*, showing an involvement of the TTSS1 apparatus in the activation of IL-8 secretion. Moreover, the use of the Δ*vp1680 *strain showed an intermediate level of IL-8 secretion when compared to the WT and *ΔvscN1 *strains, suggesting that the effector protein VP1680 is involved in the IL-8 secretion activation by the Caco-2 cells in response to the bacteria but it is not the only TTSS1 effector responsible for this activation. With the *ΔvscN2 *strain there was a higher level of IL-8 secretion by the Caco-2 cells than that observed with the WT *V. parahaemolyticus*, suggesting that TTSS2 is involved in the inhibition of the IL-8 secretion by the Caco-2 cells in response to the bacteria 24 h after the addition of the bacteria.

These results demonstrate that *V. parahaemolyticus *actively induces the transcription and production of IL-8 by the host cell. TTSS1 is involved in the activation of IL-8 production by the host while TTSS2 is involved in its inhibition. Moreover, we have demonstrated that the TTSS1 effector VP1680 is involved in the stimulation of IL-8 secretion by the host.

### The ERK signalling pathway is activated by *V. parahaemolyticus *and leads to IL-8 secretion by intestinal epithelial cells

**Figure 6:**
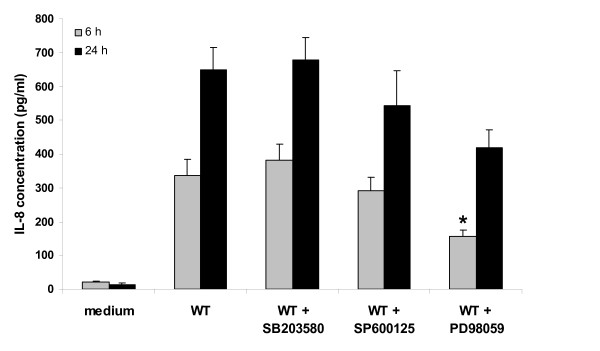
**p38 and ERK are involved in the stimulation of IL-8 secretion by *V. parahaemolyticus***. A: ELISA to detect secreted IL-8 6 h and 24 h after co-incubation with *V. parahaemolyticus *in presence of MAPK inhibitors. The experiment was performed essentially as outlined in Figure 5 with WT *V. parahaemolyticus *and the addition of MAPK inhibitors, SB203580 (5 μM), SP600125 (15 μM) or PD98059 (40 μM), as indicated. Results indicate mean ± SEM of three independent experiments. *P < 0.05 vs cells co-incubated with bacteria in absence of inhibitor.

In order to obtain a better overview of the signalling pathways leading to IL-8 activation in response to *V. parahaemolyticus*, the pharmacologic inhibitors of the MAPK signalling pathways were added during co-incubation and IL-8 secretion was quantified by ELISA (Figure 6). Addition of the inhibitors SB203580 and SP600125 had no influence on the level of IL-8 secreted by the Caco-2 cells co-incubated with WT *V. parahaemolyticus*, while the use of the ERK inhibitor PD98059 led to a significant decrease in the concentration of secreted IL-8. In fact a decrease of about 25% was seen in the IL-8 level secreted by the Caco-2 cells co-incubated with the WT *V. parahaemolyticus *when the cells have been pre-treated with PD98059. This result suggests that the inhibition of ERK signalling leads to inhibition of the resulting IL-8 secretion level. ERK signalling is a major signalling pathway activated by the WT *V. parahaemolyticus *and leads to the activation of IL-8 secretion by the eukaryotic cells.

## Discussion

The results of this study demonstrate that *V. parahaemolyticus *causes activation of MAPK in human intestinal epithelial cells and that this activation is linked to the cellular responses elicited by this bacterium. *V. parahaemolyticus *induced activation of each of the MAPK - JNK, p38 and ERK - in Caco-2 and HeLa cells (Figure 1 and 2). A mutant strain with a non-functional TTSS1 (Δ*vscN*1) did not cause MAPK activation, providing the first evidence that TTSS1 is responsible for the activation of MAPK in epithelial cells in response to infection with *V. parahaemolyticus *(Figure 2). While the role of TTSS1 in ERK activation was difficult to observe in Caco-2 cells, differences in the activation of ERK in HeLa cells co-incubated with WT compared to Δ*vscN*1 bacteria were clearly evident. *V. parahaemolyticus *therefore now joins a select group of gram-negative pathogens that use TTSS effectors to activate MAPK signalling to promote pathogen infection. Given the important role MAPK play in controlling host innate immune responses and cell growth, differentiation and death, they are commendable targets for pathogenic effectors. While several pathogens use their TTSS to inhibit MAPK activation [34,35,42,43], others activate them. For example, the inflammatory responses induced by the TTSS effectors of *Salmonella *typhimurium are related to activation of all MAPK, especially p38 which induces IL-8 secretion from epithelial cells [39], and *Burkholderia pseudomallei *utilizes its TTSS to induce IL-8 secretion and to increase bacterial internalization via activation of p38 and JNK in epithelial cells [44].

Several *Vibrio s*pp. manipulate MAPK signalling pathways to induce host cell death or disturb the host response to infection [40,45-49]. *Vibrio vulnificus *triggers phosphorylation of p38 and ERK via Reactive Oxygen Species in peripheral blood mononuclear cells thereby inducing host cell death [46]. The CtxB cholera toxin from *Vibrio cholerae *down-regulates p38 and JNK activation in macrophages leading to suppression of production of TNFα and other pro-inflammatory cytokines [40,47]. Additionally Flagellin A from *V. cholerae *contributes to IL-8 secretion from epithelial cells through TLR5 and activation of p38, ERK and JNK [48]. Despite the fact that *V. parahaemolyticus *possesses flagellin proteins similar to those of *V. cholerae *[49], cells co-incubated with heat-killed *V. parahaemolyticus *did not exhibit MAPK phosphorylation (Figure 1), suggesting an absence of TLR5 recognition of flagellin. TLR5 is activated by dissociated flagellin monomers and the sheathed *Vibrio *flagella present on intact bacteria have a limited ability to trigger host innate immunity [50]. In our studies bacteria were washed before addition to the cells and were treated at a temperature unlikely to dissociate flagellin monomers [50], thereby minimising the amounts of flagellin monomers present to trigger TLR5.

The results obtained from LDH assays, MTT assays and fluorochrome staining confirmed that the TTSS1 of *V. parahaemolyticus *is essential for the cytotoxicity of this bacterium towards epithelial cells (Figure 3). Furthermore these results show that there was no cell death detected prior to the 2 h time point, by which time MAPK activation was observed. It has been reported that undifferentiated Caco-2 cells are more susceptible than other cell types (e.g. HeLa cells) to a TTSS2-mediated delayed cytotoxicity [15,51]. While TTSS1 was required for cytotoxicity during the first 4 h of co-incubation, there was little difference in the levels of cytotoxicity observed with ΔTTSS1 bacteria compared to WT *V. parahaemolyticus *when co-incubations were performed for 6 h [51]. This delayed cell death was attributed to the VopT TTSS2 effector [51]. Delayed cytotoxicity was also observed by Burdette et al. in HeLa cells infected with ΔTTSS2/Δ*vp1680 *bacteria [29]. The mechanism of this delayed cytotoxicity is unknown. With extended co-incubations of 8 h we too saw delayed TTSS1- and VP1680-independent cytotoxicity with differentiated Caco-2 cells (unpublished data Finn and Boyd). The delayed cytotoxicity was the not the subject of this study.

The VP1680 effector protein is responsible for the TTSS1-dependent autophagic cytotoxicity against HeLa cells [25,29]. Our results demonstrated that VP1680 is required for the induction of JNK and p38 phosphorylation in Caco-2 cells (Figure 2) and that JNK and ERK, but not p38, are involved in the TTSS1-dependent cytotoxicity (Figure 4). Each of the 3 MAPK has been proposed to regulate autophagy and/or autophagic cell death, though the role and relative importance of each one seems to be dependent on cell type and on the induction stimulus [52-54]. The activation of JNK and ERK by VP1680 seems to be important for the cytotoxicity of *V. parahaemolyticus *towards epithelial cells, whereas phosphorylation of p38 by this effector protein plays a different role in modification of host cell behaviour that remains to be defined. In HeLa cells VP1680 is responsible for the activation of ERK, but plays a lesser role in the activation of JNK and p38 than it does in Caco-2 cells (Figure 2). As activation of all three MAPK in HeLa cells in response to *V. parahaemolyticus *is TTSS1-dependent, but not VP1680-dependent, this points to the existence of an additional MAPK-activating TTSS1 effector that acts in this cell line. Since VP1680 is the principal TTSS1 effector activating MAPK in Caco-2 cells, this would suggest differing sensitivities of cell lines to the TTSS effectors.

The observation that VP1680 induces phosphorylation of all 3 MAPK raises the possibility that this protein may not target the MAPK directly, but may trigger an upstream kinase. In contrast to VP1680, the VopA TTSS2 effector has been found to inhibit MAPK in macrophages by acetylating the upstream MAPK Kinase (MKK) [18,30]. It is important to note that the VopA studies were performed with transfected eukaryotic cells that expressed VopA heterologously, whereas the current study assessed MAPK activation by intact *V. parahaemolyticus*. From our studies during co-incubation of *V. parahaemolyticus *with Caco-2 cells it appears that the MAPK activation of VP1680 is dominant over the inhibitory effect of VopA. *V. parahaemolyticus *may co-ordinately regulate both TTSS to achieve appropriate control of host responses.

*V. parahaemolyticus *induced IL-8 secretion in an active manner as a result of delivery of the TTSS effector proteins into host cells (Figure 5). It appears that there may be a balance between TTSS1 and TTSS2 of *V. parahaemolyticus *where TTSS1 is involved in the activation of IL-8 production by the host while TTSS2 is involved in its inhibition. This correlates with the opposing functions of the TTSS1 effector VP1680 and the TTSS2 effector VopA in activating and inhibiting MAPK phosphorylation. Interestingly, the TTSS1 effector VP1680 mutant (Δ*vp1680*) induced intermediate amounts of IL-8, suggesting an involvement of this protein in stimulating production of this chemokine, but not an absolute requirement (Figure 5). Similarly the inhibitory studies revealed that *V. parahaemolyticus *induces secretion of IL-8 partly via modulation of the ERK signalling pathway (Figure 6). The complex effect of both TTSS of *V. parahaemolyticus *on the host immune defence machinery illustrates the powerful tools the bacteria possess to gain maximum advantage from the host environment.

## Conclusions

A better understanding of the virulence mechanisms of *V. parahaemolyticus *is imperative for better diagnosis, treatment and prevention of gastrointestinal infections. The findings presented here provide new insights into the roles of TTSS1 and TTSS2 in modulating epithelial cell responses to infection. *V. parahaemolyticus *induced JNK, ERK and p38 activation in human epithelial cells. TTSS1, and the TTSS1 effector VP1680, were of key importance for sabotaging normal MAPK cellular processes and disrupting host responses to infection. MAPK activation was associated with the cytotoxic effects exerted by the bacterium and with the induction of IL-8 secretion. The diverse roles of MAPK signalling during infection with *V. parahaemolyticus *indicate it is a significant mechanism to promote virulence.

## Methods

### Cells and reagents

*V. parahaemolyticus *RIMD2210633, O3:K6 serotype (wild type, WT) [12] was used for the construction of deletion mutants as well as to perform all experiments. The following bacterial mutants were used: VVN1 (ΔTTSS1 with non-functional TTSS1 due to deletion in the *vscN1 *gene encoding the ATPase for TTSS1), VVN2 (ΔTTSS2 with non-functional TTSS2 due to deletion in the *vscN2 *gene encoding the ATPase for TTSS2) and VVE1 (Δ*vp1680*). Bacteria were cultured at 37°C in Luria-Bertani medium supplemented with 3% (w/v) NaCl (LBN) and the addition of 1.5% (w/v) agar where appropriate. The human epithelial intestinal Caco-2 and cervical HeLa cell lines were obtained from the DSMZ (German Collection of Microorganisms and Cell Cultures). Caco-2 cells were grown as a monolayer in Dulbecco's Modified Eagle's Medium (DMEM) supplemented with 2 mM L-glutamine (Gibco), Pen-Strep (100 units/ml penicillin, 100 μg/ml streptomycin, (Gibco), 1% non-essential amino acids (Gibco) and 20% (v/v) Foetal Bovine Serum (Gibco) at 37°C, 5% CO_2_. All materials used were purchased from Sigma, unless otherwise stated. Measurement of absorbance of samples in 96-well plates was performed using a Tecan Sunrise and Magellan software.

### Construction of deletion mutant strains

Molecular biology techniques were performed according to Sambrook and Russell [55]. PCR reagents were obtained from Bioline, DNA purification kits and molecular biology enzymes from Promega and oligonucleotides from MWG/Eurofins. The standard PCR reaction volume was 50 μl, containing 50 ng template DNA, 400 nM each primer and 1× Polymerase Mix (Bioline). 1^st ^round PCR reactions in the overlap extension method were performed with Accuzyme polymerase and the standard PCR conditions were 3 min at 95°C (1 cycle), 30 sec at 95°C, 30 sec at 58°C, 2 min/kb at 68°C (30 cycles), 5 min at 68°C (1 cycle). Other PCR reactions were performed with Taq polymerase, and an extension time and temperature of 30 sec/kb and 72°C, respectively. In some cases the annealing temperature was optimised for a specific PCR reaction.

In-frame deletion mutations were constructed in the *vscN g*enes of each of the *V. parahaemolyticus *TTSS in order to inactivate each of these secretion systems independently. As the *vscN *gene encodes the ATPase that powers the secretion process, mutation of this gene eliminates secretion. The TTSS1-associated VscN1 is encoded by *vp1668 *and TTSS2-associated VscN2 is encoded by *vpa1338*. Each mutant allele was constructed by overlap PCR. The primers PrAB49 (AACGCGAACGCCACCGTC), PrAB50 (TCTGCTACGCGCTGCTTGAGC), PrAB51 (ACTTGCAGACAACTCTCCAACGCGTAC) and PrAB52 (GGAGAGTTGTCTGCAAGTCGAGTGATG) were used for generation of the *vscN1*_Δ142-1065 _allele encoding VscN1_Δ51-355_. Primers PrAB45 (GCCATCAGGTCAAGTGCAAG), PrAB47 (TCTATAGCTATTTCACCGCGGATTCTC), PrAB48 (CGGTGAAATAGCTATAGAACGCTACCC) and PrAB59 (GTCTACCGTATCTCGAATGAATAGCG) were employed to generate the *vscN2*_Δ132-1154 _allele encoding VscN2_Δ45-385_. The PCR products were cloned into pCR2.1 by TA topoisomerase cloning according to the manufacturer's instructions (Invitrogen). The alleles were then transferred into the suicide vector pDS132 [56] by extraction with the restriction enzymes *Sac*I and *Xba*I, for *vscN1 *and *vscN2 *respectively, followed by ligation into the corresponding restriction sites of pDS132. This resulted in plasmid pABGA11 containing the *vscN1*_Δ142-1065 _allele and pABGA13 containing the *vscN2*_Δ132-1154 _allele.

Triparental conjugations with *Escherichia coli *CC118λpir(pEVS104) [57] were performed to introduce pABGA11 and pABGA13 into *V. parahaemolyticus *RIMD2210633 and selection of first recombinants was performed on LBN agar containing 5 μg/ml chloramphenicol. Subsequently second recombinants were selected on LBN agar containing 10% sucrose and then screened by PCR with primers PrAB49 and PrAB50 for *vscN1 *and primers PrAB45 and PrAB59 for *vscN2*. Bacteria that contained the gene of the expected shortened length were designated VVN1 for the *vscn1 *mutant strain and VVN2 for the *vscn2 *mutant strain.

*V. parahaemolyticus *VVE1 containing a mutated *vp1680 *gene was constructed in a similar manner utilising primers PrAB88 (AAACATGGCACTGTAAGCGTCG), PrAB89 (GGTTAGCGCACTCAAGCAAATGCTTGGC), PrAB91 (GCGCGTAAGAGGCTTAGAGC) and PrAB92 (GCTTGAGTGCGC*T*AACCTAAGCAAACTTG) to remove nucleotides 161-1120. In addition a TAA stop codon was introduced at codon 51 (altered nucleotide shown in italics) so that a truncated protein would be produced.

### *V. parahaemolyticus *and epithelial cell line co-incubation studies

All experiments with Caco-2 cells were carried out on differentiated cells obtained by culturing of the cells for 7 days (3 days post-confluency). HeLa cells were seeded the day prior to the co-incubation. During co-incubations with bacteria the cells were maintained in growth medium free of Pen-Strep antibiotics. Bacteria were cultured to obtain cells in mid-log phase of growth and then washed with PBS. Monolayers were co-incubated with WT *V. parahaemolyticus *and constructed deletion mutants at an MOI of 10. After the co-incubation period samples were taken for analysis. Preliminary experiments were performed with a range of MOI. Cells infected with an MOI of 10 displayed reproducible and reliable MAPK activation and cell lysis data and so this MOI was selected for use throughout these studies. In some experiments MAPK inhibitors were added to the cells 2 h prior to the addition of the bacteria at these concentrations: 15 μM SP600125, 5 μM SB203580 and 40 μM PD184352.

### Lactate Dehydrogenase (LDH) assay

The Caco-2 cells were co-incubated with bacteria for 1, 2, 3 or 4 h. The LDH assay was performed using the CytoTox 96 Non-Radioactive Cytotoxicity Assay kit (Promega) according to the manufacturer's instructions. The results obtained were analyzed using the formulas provided by manufacturer and expressed as percentage cell lysis.

### MTT (3-(4,5-Dimethylthiazol-2-yl)-2,5-diphenyltetrazolium bromide) assay

The Caco-2 cells were co-incubated with bacteria for 1, 2, 3 or 4 h. The cells were washed and resuspended first in fresh complete medium containing 50 μg/ml gentamicin for 1 h and then 5 μg/ml gentamicin for 20 h to kill extracellular bacteria. Monolayers were then incubated in MTT solution (5 mg/ml; 50 μl/well) for a further 3 h. The medium containing MTT was removed and the insoluble violet formazan crystals were dissolved in dimethyl sulfoxide. The absorbance was measured at λ550-590 nm. Cell viability was calculated as a percentage of the untreated Caco-2 cells.

### Phase contrast light microscopy and fluorescent microscopy

The Caco-2 cells were co-incubated with bacteria for 2 and 4 h. After the co-incubation monolayers were washed and imaged by phase contrast light microscopy on a Leica DM IL inverted microscope fitted with a DFC420C digital camera using LAS software. For fluorescent microscopy after the co-incubation periods all detached and adherent Caco-2 cells were harvested, washed and stained with 230 μM propidium iodide/300 μM Hoechst 33342 for 5-10 min. Three hundred Caco-2 cells were analyzed and scored under the Olympus fluorescent microscope IX51 using Cell software and the DAPI filter (λ488 nm, Hoechst 33342 and PI positive) and the TxRed filter (λ520 nm, PI positive only).

### Immunoblotting

Following co-incubation with bacteria the epithelial cells were washed in PBS and lysed with Laemmli sample buffer. Samples were resolved on Sodium Dodecyl Sulphate Polyacrylamide Gel Electrophoresis (SDS-PAGE) and transferred to nitrocellulose. The membranes were incubated first with the following primary rabbit antibodies - phospho-SAPK/JNK (Thr183/Tyr185) mAb, phospho-p42/44(Thr202/Thr204) pAb, phospho-p38 (Thr180/Tyr182) pAb obtained from Cell Signalling Technology Inc - and then with Horse Radish Peroxidase (HRP)-conjugated anti-rabbit IgG antibody (Jackson ImmunoReseach Laboratories). Blots were developed using the enhanced chemiluminescence detection method. Non-saturated film exposures were digitized by flatbed scanning and quantified by densitometry. To detect total level of protein the membrane was re-probed with corresponding primary antibody: pan-JNK, p38 or p42/44 mouse mAb (R&D Systems).

### Cell-Based Monodansylcadaverine (MDC) Assay

Caco-2 cells were seeded 24 h prior to the addition of the chemical MAPK inhibitors. Following 2 h incubation, WT *V. parahaemolyticus *was added to each well for 3 h. The MDC assay was performed using the Autophagy/Cytotoxicity Dual Staining Kit (Cayman Chemical Company) according to the manufacturer's instructions. Incubation steps were carried out in the dark. All centrifuge steps were omitted. The results obtained were analyzed using a Leica DMI3000B microscope and Leica application suite V3.3.0 software.

### ELISA

After co-incubation of the differentiated Caco-2 monolayers with *V. parahaemolyticus*, or 20 ηg/ml IL-1β as a positive control, IL-8 in the growth medium was detected by ELISA using the Bender Medsystem human IL-8 ELISA Kit following the manufacturer's instructions. This detection of IL-8 was performed 6 h and 24 h after a 2 h co-incubation period which had been stopped by three successive washes with PBS and the addition of complete growth medium containing 50 μg/ml gentamicin.

### RNA extraction and reverse transcription PCR

RNA was extracted by the Trizol method (Invitrogen). The RNA preparations were treated with DNAse I (Invitrogen) to remove contaminating DNA and reverse-transcribed by use of an M-MLV reverse transcriptase (Invitrogen). Then PCR was performed for 30 cycles at 95°C, 30 s; 55°C, 30 s; 72°C, 30 s with a final amplification for 5 min at 72°C. The IL-8 gene was amplified using the primers IL-8 Forward GTTCCACTGTGCCTTGGTTT and IL-8 Reverse ACACAGCTGGCAATGACAAG, and the *β*-actin gene as control was amplified using β-actin Forward AAATCTGGCACCACACCTTC and β-actin Reverse AGTGGGGTGGCTTTTAGGAT. Visualisation of the PCR products was performed following agarose gel electrophoresis using SYBRsafe (Invitrogen) and a UV light source on a G:Box from SynGene and using the software GeneSnap from Syngene. Quantification was performed by comparing the intensity of the PCR product bands to the Quantitative Hyperladder I (Bioline) as a reference and then determining the ratio between IL-8 and *β-*actin PCR products in each sample.

### Statistical analysis

Significance of the differences between groups was assessed using one way analysis of variance (ANOVA) with post-hoc Tukey-Kramer multiple comparisons test using GraphPad Instat software. p < 0.05 were considered statistically significant.

## Authors' contributions

KMW carried out immunoblotting and cytotoxicity assays, participated in mutant construction and drafted the manuscript. RF carried out immunoblotting and cytotoxicity assays and participated in mutant construction. AM carried out the ELISA and RT-PCR experiments. COB participated in the design and coordination of the study. AWB participated in the design and coordination of the study. EC participated in the design and coordination of the study and hosted training visits of researchers. AB conceived of the study, participated in its design and coordination and helped to draft the manuscript. All authors read and approved the final manuscript.

## Supplementary Material

Additional file 1 **Figure S1: Morphological changes induced in Caco-2 cells by *V. parahaemolyticus *Δ*vp1680***. Caco-2 cells were co-incubated with *V. parahaemolyticus *WT, Δ*vscN1*, Δ*vscN2 *or Δ*vp1680 *for 4 h. Morphological changes of the cells were then observed by phase contrast light microscope (magnification 400×). Click here for file
