# Supplementary figures and images for: The Vibrio parahaemolyticus Type III Secretion Systems manipulate host cell MAPK for critical steps in pathogenesis
Source: BMC Microbiol. 2010 Dec 30;10:329. doi: 10.1186/1471-2180-10-329 (PMC3022711; doi:10.1186/1471-2180-10-329)

WT

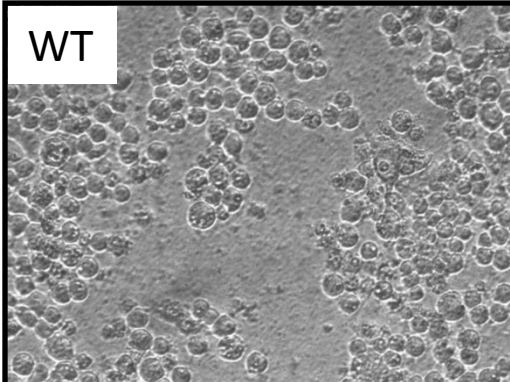

$\Delta vscN1$

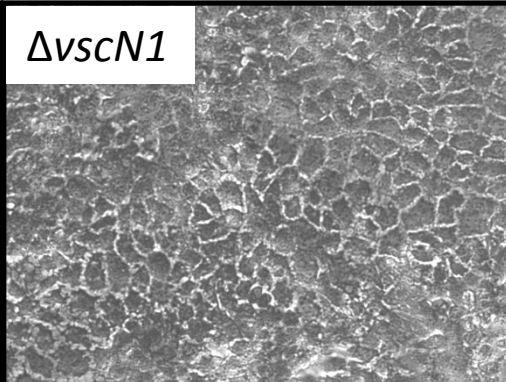

medium

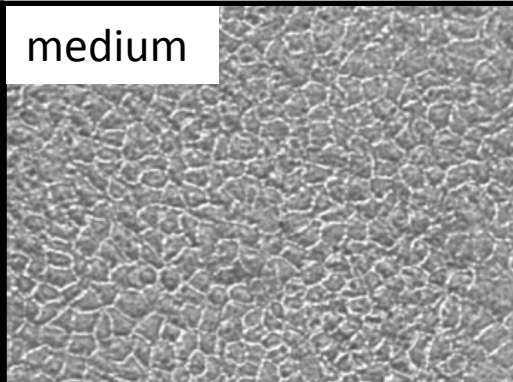

$\Delta vscN2$

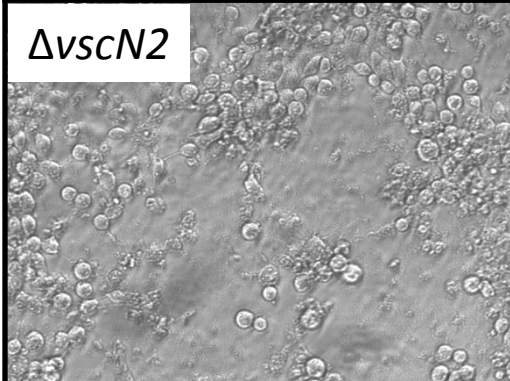

$\Delta vp1680$

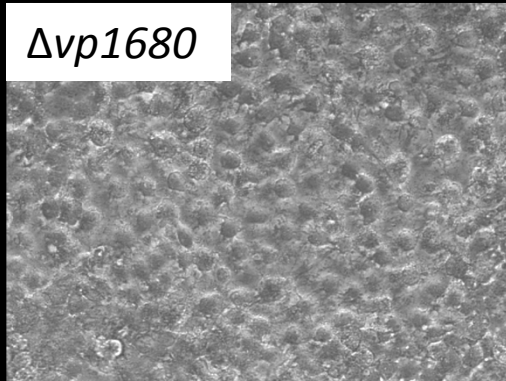

Supplement: Additional file 1 — Figure S1: Morphological changes induced in Caco-2 cells by V. parahaemolyticus Δvp1680. Caco-2 cells were co-incubated with V. parahaemolyticus WT, ΔvscN1, ΔvscN2 or Δvp1680 for 4 h. Morphological changes of the cells were then observed by phase contrast light microscope (magnification 400×). [file 1471-2180-10-329-S1.PDF]
